# Relationship between Nutrient Intake and Human Gut Microbiota in Monozygotic Twins

**DOI:** 10.3390/medicina57030275

**Published:** 2021-03-16

**Authors:** Natsuko Matsumoto, Jonguk Park, Rie Tomizawa, Hitoshi Kawashima, Koji Hosomi, Kenji Mizuguchi, Chika Honda, Ritsuko Ozaki, Yoshinori Iwatani, Mikio Watanabe, Jun Kunisawa

**Affiliations:** 1 Department of Clinical Laboratory and Biomedical Sciences, Division of Health Sciences, Osaka University Graduate School of Medicine, Osaka 565-0871, Japan; creamcollon0509@gmail.com (N.M.); ritsumiff1103@docomo.ne.jp (R.O.); nabe@sahs.med.osaka-u.ac.jp (M.W.); 2 Laboratory of Bioinformatics, Artificial Intelligence Center for Health and Biomedical Research, National Institutes of Biomedical Innovation, Health and Nutrition, Osaka 567-0085, Japan; jonguk@nibiohn.go.jp (J.P.); hkawashi@nibiohn.go.jp (H.K.); kenji@nibiohn.go.jp (K.M.); 3 Center for Twin Research, Osaka University Graduate School of Medicine, Osaka 565-0871, Japan; hosomi@nibiohn.go.jp (K.H.); honda-ch@sahs.med.osaka-u.ac.jp (C.H.); iwatani@sahs.med.osaka-u.ac.jp (Y.I.); kunisawa@nibiohn.go.jp (J.K.); 4 Laboratory of Vaccine Materials, Center for Vaccine and Adjuvant Research and Laboratory of Gut Environmental System, National Institutes of Biomedical Innovation, Health and Nutrition, Osaka 567-0085, Japan; 5 Institute for Protein Research, Osaka University, Osaka 565-0871, Japan

**Keywords:** monozygotic twins, gut microbiome, nutrients

## Abstract

*Background and Objectives*: The gut microbiota is associated with human health and dietary nutrition. Various studies have been reported in this regard, but it is difficult to clearly analyze human gut microbiota as individual differences are significant. The causes of these individual differences in intestinal microflora are genetic and/or environmental. In this study, we focused on differences between identical twins in Japan to clarify the effects of nutrients consumed on the entire gut microbiome, while excluding genetic differences. *Materials and Methods*: We selected healthy Japanese monozygotic twins for the study and confirmed their zygosity by matching 15 short tandem repeat loci. Their fecal samples were subjected to 16S rRNA sequencing and bioinformatics analyses to identify and compare the fluctuations in intestinal bacteria. *Results*: We identified 12 genera sensitive to environmental factors, and found that *Lactobacillus* was relatively unaffected by environmental factors. Moreover, we identified protein, fat, and some nutrient intake that can affect 12 genera, which have been identified to be more sensitive to environmental factors. Among the 12 genera, *Bacteroides* had a positive correlation with retinol equivalent intake (*rs* = 0.38), *Lachnospira* had a significantly negative correlation with protein, sodium, iron, vitamin D, vitamin B6, and vitamin B12 intake (*rs* = −0.38, −0.41, −0.39, −0.63, −0.42, −0.49, respectively), *Lachnospiraceae* ND3007 group had a positive correlation with fat intake (*rs* = 0.39), and *Lachnospiraceae* UCG-008 group had a negative correlation with the saturated fatty acid intake (*rs* = −0.45). *Conclusions*: Our study is the first to focus on the relationship between human gut microbiota and nutrient intake using samples from Japanese twins to exclude the effects of genetic factors. These findings will broaden our understanding of the more intuitive relationship between nutrient intake and the gut microbiota and can be a useful basis for finding useful biomarkers that contribute to human health.

## 1. Introduction

The human microbiota consists of over 100 trillion microbes with over 1000 species in the gut [1], which comprise the gut microbiota. The human gut microbiota has been an active field of research as it is closely related to various human physiological functions through the control of immune systems and metabolic functions [2,3], and closely associated with many diseases including obesity, diabetes, colorectal cancer, arteriosclerosis, and inflammatory bowel disease. 

Dysbiosis, which causes oxidative stress, overexpresses nitric oxide (NO), and production of NO is due to the production of reactive oxygen species (ROS) such as superoxide anion and hydrogen peroxide. It has harmful effects on human health, including inflammatory reaction.

Therefore, it is imperative to enhance our knowledge about the relationship between human gut microbiota and human health. Among the various environmental factors affecting the gut microbiota, the lifestyle factors that affect the composition of the gut microbiota are diet [4,5,6] and stress, physical activity [7], drug intake, alcohol drinking, and smoking habits [8]. Besides there are other influences such as heredity [9], genetic variation [10], parturition style, geographic effect [11], age [12], virome [13]. 

The main effect of improving the intestinal environment was to improve constipation and diarrhea and to prepare the intestines, at present, people are highly interested in diets containing probiotics and prebiotics with the aim of improving lifestyle-related disease, immune regulation, and brain function.

Gut microbiota is known to affect metabolic regulation with food and drink intake. For example, an important mechanism of metabolic regulation by the gut microbiota is the production of short-chain fatty acids (SCFA). It acts as a supplemental nutrient and specific ligand for two G protein-coupled receptors (GPCRs), targeting the gut, brain, liver, and adipose tissue and regulating appetite, energy expenditure, obesity, and glucose production [14]. 

However, it is difficult to ingest due to its odor, taste, and absorbency. Therefore, it is necessary to ingest foods rich in oligosaccharides and dietary fiber and specific Bifidobacteria capable of fermenting them [15]. 

Nutrient intake is considered to be the most important factor in the formation of the gut microbial community [16]. A previous study indicated that longitudinal dietary control changes enterotypes [17]. Another study demonstrated that varied dietary nutrient intake affects the gut microbiota composition [18], and dietary changes affect the abundance of gut microbiota in healthy adults in host-microbial interactions [19]. For these reasons, many studies are being conducted to understand the relationship between dietary nutrient intake and human gut microbiota, but due to individual differences in human gut microbiota, this relationship remains still unclear. 

All human phenotypes such as susceptibility to disease, abilities, personality, and other individual characteristics are influenced by genetic and environmental factors. The gut microbiota composition is also known to be affected by genetic and environmental influences; a previous study showed that genetic factors significantly affect some intestinal bacteria [20]. 

Therefore, we devised a study of monozygotic (MZ) twins that could assess the environmental impact on the gut microbiota after controlling for the effects of genetic diversity. The first advantage of the MZ twin study is that we can understand the difference due to environmental factors by comparing changes between twins because MZ twins share 100 % of their genetic background and common environmental factors such as intrauterine environment and domestic settings. We regard twin research as an effective way to accurately evaluate the relationship between nutrient intake and gut microbiota. In previous studies of twins, there are results that the concordance rate for the methanogen *Methanobrevibacter smithii* was higher in MZ than in dizygotic (DZ) twin pairs [20], and that the nodes of the phylogeny with the strongest heritabilities lie within the *Ruminococcaceae* and *Lachnospiraceae families*, and the *Bacteroidetes* are mostly environmentally determined [20].

Japanese people are known to live long and have unique gut microbiota compositions compared to other countries [21]. Our study is the first to focus on the relationship between human gut microbiota and nutrients using samples from Japanese twins.

In this study, we proposed a study design to exclude genetic diversity using MZ twins. Besides, bacteria that were greatly affected by environmental factors in the gut microbiota were identified, and the dietary nutrients that affected these bacteria were investigated.

## 2. Materials and Methods

### 2.1. Subjects

Healthy Japanese MZ twins were recruited from the registry established by the Center for Twin Research, Osaka University Graduate School of Medicine, and informed consent was obtained from all 56 individuals (28 MZ pairs) analyzed this study. Zygosity of subjects was confirmed by matching 15 short tandem repeat loci using the PowerPlex 16 System (Promega, Madison, WI, USA). This study was approved by the Ethics Committee of Osaka University (696-7; 17 December 2020 and 16129-8; 1 February 2021) and the National Institutes of Biomedical Innovation, Health and Nutrition, and was conducted in accordance with their guidelines (180-01; 29 May 2019 and 128-03; 25 July 2018).

### 2.2. Nutrition Data Collection

Five major nutrients, such as Protein, Fat, Carbohydrate, Mineral, and Vitamin intake were calculated from the results of a survey using a brief-type self-administered diet history questionnaire (BDHQ), which showed reasonable validity for estimating food intake [22], and these data of two members of a twin pair were collected at the same time as the fecal samples were obtained.

### 2.3. Fecal Sample Collection

Fecal samples were placed in 15 mL vials containing 3 mL guanidine thiocyanate solution (TechnoSuruga Laboratory, Shizuoka, Japan), mixed well, and stored at 4 °C until DNA extraction.

### 2.4. DNA Extraction and 16S rRNA Gene Amplicon Sequencing

The fecal sample mixtures were mechanically disrupted using the bead-beating method. DNA was extracted using a Gene Prep Star PI-80X device (Kurabo Industries, Tokyo, Japan). After DNA extraction, the V3-V4 region of the 16S rRNA gene was amplified and sequenced using the MiSeq system (Illumina, San Diego, CA, USA). All protocols including fecal sampling and 16S rRNA sequencing were performed as described previously by Hosomi et al. [23].

### 2.5. Bioinformatics Analysis

The obtained paired-end FASTQ data were trimmed and merged before the selection of operational taxonomic units (OTUs). OTU classification and diversity analyses were performed using the QIIME pipeline (version 1.9.1) [24]. All steps from FASTQ file trimming to gut microbiota diversity analysis were automatically performed according to previously described methods [25]. The OTUs were clustered against the SILVA 128 reference database [26] at 97% similarity using the USEARCH algorithm [27]. Taxonomic classification was performed using the SILVA 128 reference database until the genus level.

### 2.6. Statistical Analyses

#### 2.6.1. Noise Processing

For statistical analysis, 10,000 reads per sample were randomly selected. In addition, taxa with less than one read on mean were eliminated as noise; consequently, 133 genera were obtained. Dietary analysis was performed using phyloseq R package [28].

#### 2.6.2. Standardization

To standardize the comparison of the genera and avoid bias in the magnitude of the composition ratio, the original composition value (genus *X*) was assigned as follows (1) and the Q value was calculated. Standardization of taxonomy data was calculated using *data.Normalization* function in the clusterSim R package.

(1) $$Q=\frac{\mathrm{original}\;\mathrm{value}-\mathrm{mean}\;\mathrm{of}\;\mathrm{genus}\;X\;\mathrm{values}}{\mathrm{SD}\;\mathrm{of}\;\mathrm{genus}\;X\;\mathrm{values}}$$

#### 2.6.3. Extraction of the Susceptible Genera to Environmental Factors

Subsequently, the twin with a higher genus *X* composition than the other one was designated as Twin1 and the latter as Twin2, so the Twin1 and Twin2 switched places within pairs for each genus. The intra-twin difference (ITD) was calculated using the following expression (2).

(2) $$\mathrm{ITD}x=Q\;\mathrm{of}\;Twin1\left(\mathrm{higher}\;\mathrm{genus}\;X\;\mathrm{composition}\right)-Q\;\mathrm{of}\;Twin2$$

**Figure 1:**
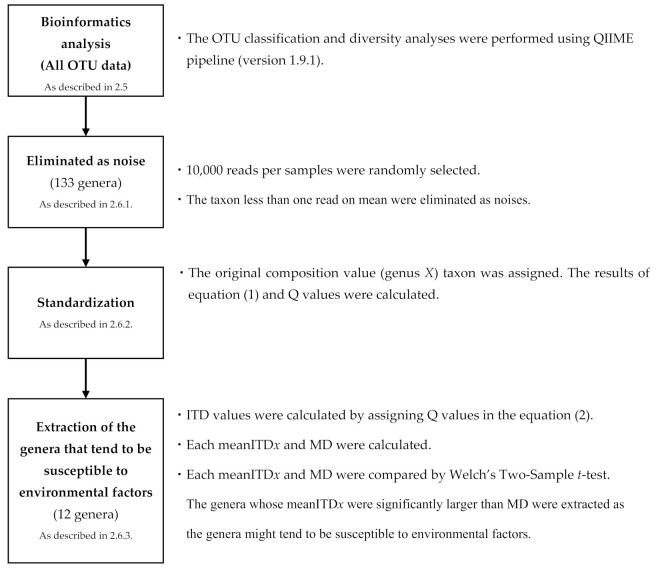
Extraction steps of susceptible genera to environmental factors. (OTU: Operational taxonomic unit, ITD: Intra-twins difference, mean ITD*x*: the mean ITD values of genus *X* for each of the 133 genera, MD: the mean of ITD values of 28 pairs for all 133 genera).

Also, the mean ITD values of genus *X* for each of the 133 genera (mean ITD*x*) and the mean ITD values of all 28 pairs for all 133 genera (MD) were calculated. Subsequently, mean ITD*x* and MD were compared using Welch’s Two-Sample *t*-test (Figure 1).

#### 2.6.4. Correlation between the Target Genera and Nutrient Intake

The genera whose mean ITD*x* were significantly larger than MD were extracted as the genera that might be susceptible to environmental factors and targeted in this analysis.

Intra-twin nutrient difference (ITND) was calculated by Equation (3) using the data from the BDHQ survey.

(3) ITND=nutrient intake of Twin1−nutrient intake of Twin2

To understand the relationship between gut microbes and diet nutrient intake, a correlation between ITND of nutrient intake and ITD of genera, which are likely subject to environmental factors, was measured using Spearman’s rank method (*cor* function in stats R package).

Statistical analysis in this study was performed using R (version 3.5.0).

## 3. Results

### 3.1. Characteristics of Participants

The characteristics of the twin samples are shown in Table 1. Healthy male MZ twins participated in our study (*n* = 56).

### 3.2. Susceptible Genera to Environmental Factors

We compared the mean intra-twin differences of the genus X for each of the 133 genera (mean ITD*x*) and the mean overall 28 pair differences for all 133 genera (MD) using Welch’s Two-Sample *t*-test. The significance level that differed from MD (=0.668) was set to *p* < 0.05, and 13 final genera were selected from 133 genera (Table 2). 

Among the 13 genera, only *Lactobacillus* had a significantly smaller mean ITD than MD and was relatively unaffected by environmental factors. Besides, *Bacteroides*, *Parabacteroides*, *Lachnospiraceae* UCG-008 group, *Lachnospiraceae* UCG-004 group, *Lachnospiraceae* ND3007 group, *Lachnospiraceae* FCS020 group, *Roseburia*, *Eubacterium hallii* group, *Lachnospira*, *Faecalibacterium*, *Ruminococcaceae* UCG-003 group, and *Gardnerella* had a significantly larger mean ITDx than MD.

### 3.3. Association of Specific Genera with Nutrient Intake

The 12 genera whose mean ITD*x* was significantly larger than MD were considered to be the genera susceptible to environmental factors. We calculated the correlation between within twin-pair differences in the relative abundances of targeted genera and nutrient intake in order to assess their relationship with eliminated genetic factors. The significance level was set to *rs* = 0.27 (*p* < 0.05) [29]. Among the 12 genera, *Bacteroides* had a positive correlation with retinol equivalent (RTE) intake (*rs* = 0.38), *Lachnospira* had a significantly negative correlation with protein, sodium, iron, vitamin D, vitamin B6, and vitamin B12 intake (*rs* = −0.38, −0.41, −0.39, −0.63, −0.42, −0.49, respectively), *Lachnospiraceae* ND3007 group had a positive correlation with fat intake (*rs* = 0.39), and *Lachnospiraceae* UCG-008 group had a negative correlation with SFA intake (*rs* = −0.45) (Table 3). *Parabacteroides*, *Lachnospiraceae* UCG-004 group, *Lachnospiraceae* FCS020 group, *Roseburia*, *Eubacterium hallii* group, *Faecalibacterium*, *Ruminococcaceae* UCG-003 group, and *Gardnerella* did not show a significant correlation with the five major nutrients (protein, sodium, iron, vitamin D, vitamin B6, and vitamin B12).

## 4. Discussion

In this study, we focused on nutrient intake as an environmental factor and investigated the relationship between nutrient intake and the human gut microbiota using samples from Japanese healthy adult MZ twins. Targeting twins is the only way to consider genetic effects. We confirmed that they had not taken the antibiotic for more than two weeks because it has been clarified that gut microbiota is affected by taken antibiotic [30,31,32], and that they did not have a habit of drinking large amounts of alcohol in consideration of the effects of habitual alcohol [33,34].

First, we estimated the genera susceptible to environmental factors by comparing the intra-twin differences for each genus with the mean of overall intra-twin differences. Among the 13 extracted genera, *Lactobacillus* showed only a small intra-twin difference, so this genus may be susceptible to genetic factors. *Lactobacillus* has been reported as a genus established in infancy [35], and it is likely one of the reasons for this result. This result is inconsistent with the genera extracted in a previous study of twins [20]. However, studies focused on the association of the genus and obesity showed the decreased abundance of *Lactobacillus* in the gut microbiota in obese subjects [36], and conversely the abundance of *Lactobacillus* [37,38]. And another showed the increased abundance of *Lactobacillus* in patients with metabolic syndrome [39,40]. As mentioned above, conclusions of previous studies on the relationship of *Lactobacillus* with metabolic syndrome and obesity are not consistent. 

For *Lactobacillus*, there are many studies on its function as a probiotic. A recent metagenomic analysis of 8-week-old Swiss mice fed a high-fat diet showed that treatment with a probiotic mixture of *Lactobacillus* and *Bifidobacterium* significantly altered the composition of the gut microbiota and increased insulin sensitivity. Showed that it was increased [41]. Probiotic *Lactobacillus* has been shown to have the potential to improve gastrointestinal barrier function through the growth of several harmful bacteria [42,43]. And probiotic *Lactobacillus* has been shown to enhance gastrointestinal barrier function by the growth of harmful bacteria in non-alcoholic fatty acid liver disease and IBD [42,44].

Furthermore, previous studies demonstrated that metabolic syndrome and obesity are influenced by genetic factors to some degree [45]. In order to clarify these relationships, genetic factors should be considered.

In our study, the other 12 genera had significantly large intra-twin differences; therefore, they may be susceptible to environmental factors.

Among the 12 genera, *Bacteroides* had a positive correlation with RTE intake (*rs* = 0.38). *Bacteroides* are known to have immunomodulatory activity on the intestinal immune system [46,47] and Type 1 diabetes [48]. In addition, *Bacteroides* can decompose indigestible oligosaccharides as nutrients and can activate their proliferation by using fructooligosaccharides as a food resource [49]. *Bacteroides* have the highest abundance among the human gut microbiota, so it may be meaningful as a target to study the gut microbiota relationship with environmental factors, including dietary intake. A previous study demonstrated that vitamin A treatment in vitamin A-reduced mice in a necrotic enterocolitis model increased the relative abundance of *Bacteroides*, which is in agreement with our results [50], however, it is unclear that certain nutrition or diets affect it in observational studies of humans. On the other hand, the threshold required to cause dysbiosis varies among the affected bacterial population. A wide range of changes in the main phyla of Bacteroides and Firmicutes may not lead to pathological consequences, but increased amounts of peripheral groups can cause havoc [51]. Enterobacteriaceae bacteria can spread rapidly following changes in the oxidative state of the intestine, such as during inflammation. Due to the febrile activity of the Enterobacteriaceae lipopolysaccharide (LPS), the growth of this bacterial family usually intensifies the ongoing inflammatory response [51].

There was a significantly negative correlation between *Lachnospira* and protein intake. A previous study demonstrated that the relative abundance of *Bacteroides* decreased with hypocaloric high-protein intake in patients with non-alcoholic fatty liver disease, which is consistent with our results of negative correlation [52]. In addition, *Lachnospira* was negatively correlated with PRT, NA, FE, VD, VB6, and VB12 intake (*rs* = −0.38, −0.41, −0.39, −0.63, −0.42, −0.49, respectively). A previous study demonstrated that the relative abundance of *Lachnospira* was positively associated with vegetable intake [53], and that *Lachnospira* had a positive correlation with stilbene in an observational study [54]. Ingestion of a high-fat diet (HFD) induces oxidative stress and microbial dysbiosis, the latter playing an important role in the development of metabolic syndrome. Polyphenol supplementation affected the gut microbiota by improving the ratio of butyric acid producers Blautia and Dorea in the Lachaospiraceae family and inhibiting the growth of disease and inflammation-related bacterial species such as *Bacteroides* and *Desulfovibrionaceaesp* [55].

*Lachnospiraceae_*ND3007 group may be affected by fat intake. There are no significant insights into the relationship between Lachnospiraceae and fat intake, but it has been reported that fat intake improves the expression of inflammatory cytokines [56]. Thus, *Lachnospiraceae* is likely an important family associated with inflammation. However, there are no previous reports on the relationship among environmental factors, *Lachnospiraceae* UCG-008 group, *Lachnospiraceae* UCG-004 group, *Lachnospiraceae* FCS020 group, *Roseburia*, and *Eubacterium hallii* group.

A higher relative abundance of *Roseburia* has been reported in active people than sedentary people [57]. We found no relationship between nutrient intake and *Eubacterium hallii* group, but it could alter the function of the gut microbiota and its metabolites may contribute to optimal metabolic function [58]. As stated above, the family Lachnospiraceae may be linked to environmental factors and is known to be able to protect against human colon cancer by producing butyric acid [59], therefore, our findings on Lachnospiraceae may be significant. *Roseburia intestinalis* and *Eubacterium hallii* metabolize dietary fiber as a major SCFA producer that provides an energy source for enterocytes and achieves anti-inflammatory effects in the intestine [60].

Administration of SCFA producer *Faecalibacterium prausunitzii* to mice fed a high-fat diet increased gastrocranial muscle mass and increased expression of the mitochondrial respiratory chain complex [61]. However, the ability of *Faecalibacterium prausnitzii* to produce SCFA has also been shown to be mediated by interaction with other microbial species, including *bifidobacteria* [62]. Modulation of SCFA metabolomics patterns may represent a breakthrough in IBD studies. Butyric acid is the target of this response because the proportions of the three major acids vary by target group and decrease as they move between segments. The role of these acids in controlling inflammatory growth increases with a decrease in oxidative stress, as well as the number of preferred strains [63].

There are no reports about relationships between vitamins and gut microbiota, but these vitamins may be targets for further investigation. In addition, knowledge about the relationship among gut microbiota, nutrient intake, and human biological functions may be useful biomarkers that can ascertain human health.

This study had some limitations. Our data may be biased because the examined sample number was not sufficient for strong statistical analysis (28 pairs). Further studies with higher number of subjects are needed in the future to confirm their relationship.

## 5. Conclusions

We examined the genetic and environmental influences on gut microbiota using an analytical method that focused on the differences within pairs of MZ twins. *Lactobacillus*, for which the difference between twins was not statistically significant, may be susceptible to genetic factors. On the other hand, it was suggested that the aforementioned 12 genera are sensitive to nutrient intake. Our results demonstrated that the susceptibility of gut microbiota to environmental factors is variable. By analyzing the differences between identical twins and eliminating genetic factors, we identified the relationship between nutrient intake and the composition of the human gut microbiota. 

## Figures and Tables

**Table 1:** Participant characteristics and mean scores of nutrient intake (*n* = 56).

		Mean	±	SD	(Min	–	Max)	Median
Age		59.3	±	19.4	20	–	80	67.5
Body mass index		23.4	±	3.9	15.5	–	32.9	23.8
Nutrients								
Energy intake	(kcal/day)	2057.5	±	587.4	858.9	–	3564.9	1946.5
Protein (PRT)	(g/1000 kcal)	38.5	±	7.4	20.3	–	54.6	37.3
Fat (FAT)	(g/1000 kcal)	30.2	±	6.6	18.5	–	46.1	30.2
Saturated Fatty Acid (SFA)	(g/1000 kcal)	7.9	±	2.1	4.6	–	14.0	7.6
Carbohydrates (CHO)	(g/1000 kcal)	133.9	±	19.7	94.7	–	172.0	135.3
Total Dietary Fiber (TDF)	(g/1000 kcal)	6.7	±	1.8	2.5	–	10.2	6.8
Sodium (Na)	(mg/1000 kcal)	2307.0	±	479.2	1300.8	–	3630.0	2271.6
Potassium (K)	(mg/1000 kcal)	1459.7	±	429.4	564.2	–	2307.9	1412.4
Calcium (Ca)	(mg/1000 kcal)	319.4	±	122.5	91.9	–	625.7	310.7
Iron (Fe)	(mg/1000 kcal)	4.5	±	1.1	2.2	–	6.7	4.7
Retinol equivalent (RTE)	(µg/1000 kcal)	434.9	±	197.6	98.2	–	907.4	390.2
Vitamin D (VD)	(µg/1000 kcal)	7.7	±	4.6	0.2	–	23.2	6.6
Alpha-tocopherol (ATC)	(µg/1000 kcal)	4.2	±	1.2	2.1	–	8.1	4.3
Vitamin K (VK)	(µg/1000 kcal)	192.1	±	91.7	33.9	–	441.9	189.9
Vitamin B1 (VB1)	(mg/1000 kcal)	0.4	±	0.1	0.2	–	0.7	0.4
Vitamin B2 (VB2)	(mg/1000 kcal)	0.7	±	0.2	0.3	–	1.3	0.7
Vitamin B6 (VB6)	(mg/1000 kcal)	0.7	±	0.2	0.3	–	1.6	0.7
Vitamin B12 (VB12)	(µg/1000 kcal)	4.9	±	2.6	0.3	–	14.2	4.3
Vitamin C (VC)	(mg/1000 kcal)	64.6	±	28.1	17.6	–	151.3	60.2

The nutrient intake scores were calculated from the results of a brief-type self-administered diet history questionnaire. SD: standard deviation.

**Table 2:** The final genera selected based on the mean intra-twin differences from 133 genera.

Family	Genus	The Mean Intra-Twin Differences (Mean ITD*x*)	*p* Value	
ALL		0.668	―	
Lactobacillaceae	*Lactobacillus*	0.380	0.015	*
Bacteroidaceae	*Bacteroides*	1.338	0.000	*
Bifidobacteriaceae	*Gardnerella*	1.073	0.035	*
Lachnospiraceae	*Lachnospiraceae* UCG-008 group	1.163	0.006	*
Lachnospiraceae	*Lachnospiraceae* UCG-004 group	1.075	0.018	*
Lachnospiraceae	*Lachnospiraceae* ND3007 group	1.063	0.022	*
Lachnospiraceae	*Lachnospiraceae* FCS020 group	1.060	0.026	*
Lachnospiraceae	*Roseburia*	1.146	0.020	*
Lachnospiraceae	*Eubacterium hallii* group	0.987	0.032	*
Lachnospiraceae	*Lachnospira*	1.037	0.041	*
Porphyromonadaceae	*Parabacteroides*	1.064	0.028	*
Ruminococcaceae	*Faecalibacterium*	1.043	0.028	*
Ruminococcaceae	*Ruminococcaceae* UCG-003 group	1.030	0.038	*

Welch’s Two-Sample *t*-test (* *p* < 0.05).

**Table 3:** Correlations between the targeted genera and nutrient intake.

Family	Genus	Protein	Fat		Carbohydrate		Mineral				Vitamin								
		PRT	FAT	SFA	CHO	TDF	NA.	K	CA	FE	RTE	VD	ATC	VK	VB1	VB2	VB6	VB12	VC
*Bacteroidaceae*	*Bacteroides*	0.11	−0.10	0.00	0.10	0.01	−0.12	−0.05	0.27	−0.06	0.38 *	0.12	−0.03	−0.07	−0.10	0.13	0.00	0.14	−0.14
*Bifidobacteriaceae*	*Gardnerella*	0.13	0.02	0.07	−0.20	−0.20	0.11	−0.14	−0.15	−0.21	−0.23	0.19	−0.24	−0.23	−0.10	−0.15	0.01	0.17	−0.33
*Porphyromonadaceae*	*Parabacteroides*	0.19	−0.21	0.01	−0.08	0.13	0.13	0.14	0.16	0.08	−0.14	0.28	0.00	−0.06	0.09	0.02	0.06	0.10	0.19
*Lachnospiraceae*	*Eubacterium hallii group*	−0.10	−0.06	−0.02	0.08	−0.15	−0.17	−0.05	0.07	−0.10	0.33	−0.21	−0.13	−0.18	−0.14	0.10	−0.24 *	−0.23	−0.08
*Lachnospiraceae*	*Lachnospira*	−0.38 *	0.11	0.14	0.22	−0.31	−0.41 *	−0.37	−0.20	−0.39 *	0.15	−0.63 *	−0.32	−0.25	−0.36	−0.10	−0.42	−0.49 *	−0.32
*Lachnospiraceae*	*Lachnospiraceae FCS020 group*	−0.14	−0.19	−0.24	0.11	−0.14	−0.26	−0.12	−0.31	−0.17	−0.31	−0.30	−0.21	0.07	−0.16	−0.18	−0.11	−0.29	0.07
*Lachnospiraceae*	*Lachnospiraceae ND3007 group*	0.16	0.39 *	0.32	−0.28	0.25	−0.04	0.12	0.24	0.16	0.02	0.00	0.15	0.32	0.22	0.12	0.11	0.03	0.16
*Lachnospiraceae*	*Lachnospiraceae UCG−004*	0.23	0.13	0.20	−0.25	0.14	0.09	0.15	0.22	0.14	0.06	0.16	0.07	0.18	0.15	0.21	0.17	0.13	−0.02
*Lachnospiraceae*	*Lachnospiraceae UCG−008*	−0.08	−0.32	−0.45 *	0.12	−0.04	−0.20	0.04	−0.10	−0.08	0.03	0.05	0.05	−0.05	−0.02	−0.23	−0.04	−0.02	0.12
*Lachnospiraceae*	*Roseburia*	0.05	−0.08	−0.15	0.01	−0.03	−0.20	−0.04	−0.07	−0.02	0.01	−0.04	−0.03	−0.01	0.03	−0.08	−0.06	−0.08	0.14
*Ruminococcaceae*	*Faecalibacterium*	−0.16	0.00	0.07	0.16	−0.14	−0.10	−0.09	0.00	−0.14	−0.14	−0.26	−0.17	−0.30	−0.17	0.02	−0.30	−0.31	−0.02
*Ruminococcaceae*	*Ruminococcaceae UCG−003*	−0.03	0.17	0.16	−0.18	0.09	0.03	0.14	−0.02	0.10	−0.18	−0.13	0.04	0.27	0.17	0.10	0.15	−0.11	0.19

Spearman’s rank method (* *p* < 0.05).

## Data Availability

Data sharing not applicable. No new data were created or analyzed in this study. Data sharing is not applicable to this article.

## Abbreviations

ITD	Intra-twins difference
mean ITD*x*	the mean ITD values of genus *X* for each of the 133 genera
MD	the mean of ITD values of 28 pairs for all 133 genera
ITND	Intra-twins nutrient difference
